# Acute Interstitial Pancreatitis With a Normal Lipase Level in the Background of Inflammatory Bowel Disease: A Case Report

**DOI:** 10.7759/cureus.16417

**Published:** 2021-07-16

**Authors:** Abubakar Tauseef, Victor Chalfant, Sunil Nair, Avdesh Buragadda, Maryam Zafar

**Affiliations:** 1 Internal Medicine, Creighton University School of Medicine, Omaha, USA; 2 Internal Medicine, Dow International Medical College, Karachi, PAK

**Keywords:** interstitial, pancreatitis, normal, lipase, inflammatory bowel disease

## Abstract

Acute interstitial pancreatitis is usually diagnosed on the basis of clinical findings, elevated lipase level, and imaging. However, herein we present a case of a 44-year-old Caucasian male who presented with pancreatitis diagnosed on the clinical grounds in the background of inflammatory bowel disease.

## Introduction

The annual incidence of acute pancreatitis accounts for approximately 13-45 cases per year for every 100,000 adults in the US population [1]. Radiologically, pancreatitis is commonly classified based on the Atlanta classification system with two broad categories: interstitial pancreatitis and necrotizing pancreatitis [2]. An important component for the management of acute pancreatitis is addressing the underlying predisposing factors as it can guide therapeutic interventions and prevent a recurrence. Alcohol abuse and choledocholithiasis account for 75-85% of cases [3]. However, there are many other causes of acute pancreatitis including trauma, metabolic disorders, neoplasms, infections, and drugs. A good history is essential for the initial evaluation of acute pancreatitis along with laboratory evaluation of liver biomarkers and triglycerides as well as imaging studies such as abdominal US or ERCP. The diagnostic accuracy with an amylase cutoff of 110 IU/L and lipase cutoff of 80 IU/L both had a negative predictive value of 92% in detecting acute pancreatitis [4]. In presumptive gallstone etiology of acute pancreatitis an ALT or AST at level three times the upper limit of normal is considered predictive [5]. Here we present an unusual case of acute pancreatitis with normal diagnostic lab values due to presumed inflammatory bowel disease etiology.

## Case presentation

A 44-year-old Caucasian man presented to the emergency department (ED) with pain in the lower abdomen for the previous 48 hours, and associated nausea and decreased appetite. He had a BMI of 21 and his past medical history consisted of hypertension on amlodipine, ulcerative colitis (UC) with complications of small bowel obstruction, past surgical history of colectomy five years back status post colorectal dysplasia. The patient reported that this was the first time that he had this episode; he denied any similar episode in the past, as well as any bowel movement or flatus passed in the last 24 hours. The patient denied associated fever, chills, vomiting, diarrhea, or hematochezia and pharmacological treatment for ulcerative colitis to date.

Physical examination was unremarkable except for a constant, non-radiating, dull abdominal pain of 8/10 in intensity, being responsive to morphine and hypoactive bowel sounds. Laboratory investigations showed hemoglobin of 15 gram/dl, white blood count of 16,800 x 103 mm3, the calcium of 9.2 mg/dL, aspartate aminotransferase of 14 U/l, alanine aminotransferase of 19 U/l, alkaline phosphatase of 81 U/l, amylase of 19 U/l, lipase of 25 U/l and triglycerides of 76 mg/dL. Based on the laboratory investigations, an ultrasound of the abdomen was ordered, the results of which were unremarkable. CT scan of the abdomen with contrast was planned to determine the cause of non-specified pain.

**Figure 1:**
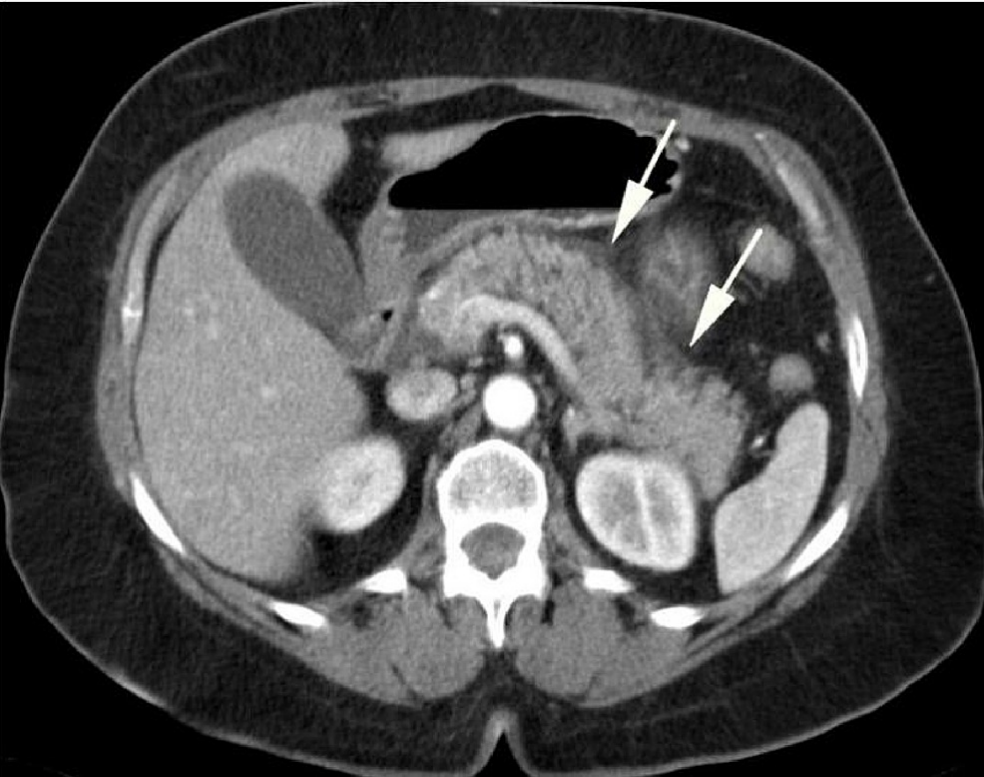
CT abdominal imaging with contrast showing acute interstitial pancreatitis (marked with an arrowhead).

The patient was started on Ringer’s lactate at a rate of 250cc/hr, morphine for pain management, and ondansetron for nausea. CT abdominal imaging with contrast revealed findings significant for acute interstitial pancreatitis, particularly of the pancreatic tail (Figure 1). The patient was diagnosed with acute pancreatitis in the background of ulcerative colitis and was continued with the same management plan. On day 3, the patient was started on a liquid diet and was discharged home with a follow-up with the primary care physician and gastroenterologist for underlying ulcerative colitis. A CT scan was planned during his follow-up visit in two weeks, which the patient did not attend.

## Discussion

Acute pancreatitis remains a major cause of hospital admissions and mortality in hospitals. According to Popa et al., the overall mortality rate of acute pancreatitis was 21.1% [6]. In recent years, acute pancreatitis has accounted for an increase in hospital volume utilization in the United States [7]. In order to improve outcomes, early fluid resuscitation within 24 to 72 hours significantly lessens complications of acute pancreatitis by reducing organ failure, admission to the ICU, and hospital length of stay [8]. 

Inflammatory bowel disease in itself represents an uncommon yet recognizable cause of acute pancreatitis in the literature [9]. Even in the absence of disease-modifying anti-inflammatory bowel disease drugs such as amino-salicylates (mesalamine) or azathioprine, patients with IBD have a higher incidence of acute pancreatitis [10]. According to Iida et al., the overall incidence of acute pancreatitis in the West has an odd rate as high as 4.3 times (3.1%) in Crohn's Disease and 2.1 times (1.2%) in ulcerative colitis compared to the general population [11]. IBD may indirectly result in acute pancreatitis due to an increased risk of extraintestinal complications such as thromboembolism [12]. Therefore, early recognition of acute pancreatitis in the differential diagnosis of intense abdominal pain is of the utmost importance. 

Although diagnostic labs have a clear value in indicating acute pancreatitis, negative labs cannot exclude a diagnosis. Based on 2006 practice guidelines from the American College of Gastroenterology, the diagnosis of acute pancreatitis requires two out of the three following features: (1) abdominal pain characteristic of acute pancreatitis, (2) serum amylase and/or lipase ≥3 times the upper limit of normal, and (3) characteristic findings of acute pancreatitis on CT scan [13]. In our case, solely the recognition of intense lower quadrant abdominal pain, not classical epigastric pain nor abnormal amylase or lipase levels, prompted a CT scan of the abdomen and urgent treatment. Although the ubiquitous use of CT exposure due to ionizing radiation has serious concerns, diagnostic imaging is indicated for unexplained acute abdominal pain [14].

## Conclusions

Even in the absence of abnormal lab values or classic epigastric abdominal pain, acute pancreatitis should be considered as a differential diagnosis in patients with IBD, especially if unexplained, and should warrant CT imaging. Early recognition and treatment of acute pancreatitis can reduce hospital complications and improve overall mortality.
